# Impact of different respiratory gating methods on target delineation and a radiotherapy plan for solitary pulmonary tumors

**DOI:** 10.1002/cam4.7322

**Published:** 2024-05-24

**Authors:** Dongping Shang, Jinghao Duan, Yong Yin, Ruozheng Wang

**Affiliations:** ^1^ Department of Radiation Oncology Shandong Cancer Hospital and Institute, Shandong First Medical University and Shandong Academy of Medical Sciences Jinan China; ^2^ Department of Radiation Oncology Affiliated Tumor Hospital of Xinjiang Medical University Urumqi China

**Keywords:** lung carcinoma, radiotherapy, respiratory gating, tumor target volume

## Abstract

**Background and Purpose:**

Respiratory movement has an important impact on the radiotherapy for lung tumor. Respiratory gating technology is helpful to improve the accuracy of target delineation. This study investigated the value of prospective and retrospective respiratory gating simulations in target delineation and radiotherapy plan design for solitary pulmonary tumors (SPTs) in radiotherapy.

**Methods:**

The enrolled patients underwent CT simulation with three‐dimensional (3D) CT non gating, prospective respiratory gating, and retrospective respiratory gating simulation. The target volumes were delineated on three sets of CT images, and radiotherapy plans were prepared accordingly. Tumor displacements and movement information obtained using the two respiratory gating approaches, as well as the target volumes and dosimetry parameters in the radiotherapy plan were compared.

**Results:**

No significant difference was observed in tumor displacement measured using the two gating methods (*p* > 0.05). However, the internal gross tumor volumes (IGTVs), internal target volumes (ITVs), and planning target volumes (PTVs) based on the retrospective respiratory gating simulation were larger than those obtained using prospective gating (group A: *p*_IGTV_ = 0.041, *p*_ITV_ = 0.003, *p*_PTV_ = 0.008; group B: *p*_IGTV_ = 0.025, *p*_ITV_ = 0.039, *p*_PTV_ = 0.004). The two‐gating PTVs were both smaller than those delineated on 3D non gating images (*p* < 0.001). V_5Gy_, V_10Gy_, V_20Gy_, V_30Gy_, and mean lung dose in the two gated radiotherapy plans were lower than those in the 3D non gating plan (*p* < 0.001); however, no significant difference was observed between the two gating plans (*p* > 0.05).

**Conclusions:**

The application of respiratory gating could reduce the target volume and the radiation dose that the normal lung tissue received. Compared to prospective respiratory gating, the retrospective gating provides more information about tumor movement in PTV.

## 1 INTRODUCTION

Radiation therapy is an important treatment for patients with non‐small cell lung cancer (NSCLC) as it helps restrict tumor growth locally and improves overall survival rates. However, the accurate targeting of tumors is challenging because of respiratory movement. During radiation therapy, the tumor and surrounding structures in the lung undergo changes in position, volume, and shape owing to respiratory motion.^1^ Conventional CT simulation images obtained using non gating 3DCT helical scanning only show the instantaneous position and shape of the tumor at a random time point in the respiratory cycle. This implies that the gross tumor volume (GTV) delineated on these images may not accurately represent the tumor movement range throughout the respiratory cycle. To compensate for this movement and ensure accurate targeting, a safety margin is typically added when delineating the planning target volume (PTV) based on 3DCT simulation images.^2^ However, this safety margin unavoidably increases the radiation dose delivered to normal tissues surrounding the tumor. Respiratory motion is thus a crucial factor affecting lung tumor radiotherapy. To achieve precise radiotherapy for lung tumors, tumor movement should be assessed accurately within the respiratory cycle. This assessment, along with individualized target delineation and radiotherapy plan design, forms the foundation for precise radiotherapy for lung tumors.^1^, ^2^, ^3^

The displacement and deformation of lung tumors caused by respiratory movement vary considerably owing to factors such as tumor location, GTV size, and staging of NSCLC.^4^, ^5^ This individual variability makes it challenging for radiation oncologists to accurately expand the safety margin and delineate the PTV based on traditional experience and documentation if they aim to cover the full range of tumor movement during the respiratory cycle.^6^, ^7^ Several researchers, including David,^8^ Li,^9^ and Guckenberger,^10^ have used different methods to measure tumor displacement caused by respiratory motion. The use of four‐dimensional (4D) cone beam CT (CBCT) technology allows the real‐time observation of tumor trajectory and displacement measurement in three dimensions. However, 4D CBCT images often contain numerous artifacts and require further refinement in terms of image quality.^8^, ^11^ Currently, respiratory gating is the predominant technology used to capture the movement information of thoracic and abdominal tumors. It is widely used in radiotherapy simulations and target delineation for these tumor types.^12^, ^13^

There are two forms of respiratory gating technology: prospective and retrospective. These two techniques differ in their image acquisition modes. Prospective gating technology uses intermittent axial scanning. The patient is instructed to breathe calmly, and when the respiratory amplitude reaches the preset amplitude threshold, the real‐time position management (RPM) system automatically triggers the simulation CT device to generate X‐rays. The CT detector then acquires tumor images at the end‐inspiration or end‐expiration states. Conversely, retrospective respiratory gating technology adopts continuous 4DCT helical scanning. A CT detector acquires tumor images while the patient breathes calmly. The detector continuously acquires images from the inspiratory peak to the expiratory trough during the respiratory cycle. These images also capture tumor movement caused by baseline drift between different respiratory cycles.^14^

In this study, prospective and retrospective respiratory gating techniques were used for radiotherapy tumor simulation, target delineation, and plan design. We compared the differences in tumor target volumes and irradiation doses to normal lung tissue based on these two respiratory gating techniques. The objective of this study was to evaluate the application value of both gating techniques in target delineation and radiotherapy plan design for solitary pulmonary tumors (SPTs).

## 2 MATERIALS AND METHODS

### 2.1 Patient selection

This retrospective analysis was approved by the ethics board of Shandong Cancer Hospital and Institute, and the need for informed consent from patients was waived. Twenty‐four patients with SPTs who underwent stereotactic body radiotherapy (SBRT) at Shandong Cancer Hospital from October 2018 to December 2020 were included in this study. The diagnosis of these tumors was confirmed either pathologically or clinically. The cases included 12 cases of adenocarcinoma, five cases of squamous cell carcinoma, and seven cases of lung metastases. The patients were divided into two groups, A and B, based on tumor location. Group A comprised five cases with tumors in the right upper lobe and six cases with tumors in the left upper lobe. Group B comprised six cases with tumors in the right middle‐lower lobe and seven cases with tumors in the left lower lobe. The inclusion criteria were as follows: (1) peripheral lung tumors or metastases; (2) solitary tumor without adhesion to the pleura; and (3) ability to cooperate during the simulation with prospective and retrospective respiratory gating in the calm breathing state.

### 2.2 Simulation and image acquisition

Patients were immobilized in the supine position with their arms raised above their heads using individualized vacuum bags. A CT simulation was performed using a Philips Brilliance Big Bore CT scanner. CT scanning parameters were set as follows: 120 kV, 200 mAs, a tube rotation period of 0.75 s per cycle, and a 16‐row × 1.5 mm detector combination. The image layer thickness and spacing were both set to 3 mm, and the reconstruction matrix was 512 × 512. To ensure consistency in the respiratory amplitude and breathing rate, the patients were provided respiratory training before the CT simulation. This training helped reduce image artifacts caused by abnormal physiological activities such as coughing or deep breath‐holding during the simulation. Each patient's respiratory motion was monitored using the Varian RPM system, which uses an external surrogate to track respiratory motion. A marker box with neon circular markings was placed near the diaphragm, where the amplitude of breath movement was most significant. An infrared camera attached to the end of the CT table monitored the motion of the marker box and generated respiratory waves. This respiratory wave was transmitted to a Big Bore CT device for the gating simulations. For prospective gating, the CT machine performed axial scanning triggered by a respiratory signal. The RPM system triggered the CT equipment at the crest and trough of the respiratory signals. Two sets of axial CT images were acquired during the end‐inspiration and end‐expiration periods. Retrospective gating involved small‐pitch 4DCT helical scanning and continuous scanning of the respiratory cycle. The detector collected volume data, which were then reconstructed into CT images in ten different phases (0%, 10%, 20%, …, 90%) based on the respiratory phase.^15^ Finally, helical scanning of the region of interest was completed while the patient held their breath.

### 2.3 Target delineation and radiotherapy plan design

Conventional non gating 3DCT images, CT images collected using two gating methods, and the breath‐holding state were relayed to the treatment planning system. To ensure the accuracy of target area delineation and eliminate errors caused by different observers, the same experienced oncologist contoured the target area and organs at risk (OAR) under the same window width and window level (WW: 1600, WL:−600). The delineations of the target area and OAR were reviewed and confirmed by a senior oncologist. The GTV_con_ was constructed based on 3DCT images under conventional non gating conditions. According to the pathological type of tumor, adenocarcinomas, squamous cell carcinomas, and metastases were expanded to 8 mm, 6 mm, and 5 mm subclinical lesion areas, respectively, to form CTV_con_, which was constructed using appropriate corrections to avoid OAR. CTV_con_ expanded the safety margin by 5 mm in the left–right and anterior–posterior directions, the upper lobe tumor by 10 mm in the superior–inferior direction, and the safety margin by 15 mm in the superior–inferior direction for middle‐lower lobe tumors to construct PTV_con_ ^16^ GTV_in_ and GTV_ex_ were constructed based on end‐inspiratory and end‐expiratory CT images obtained by prospective gating simulation, respectively. Following this, GTV_in_ and GTV_ex_ were combined to derive the internal gross tumor volume (IGTV_pro_). GTV_0%_, GTV_10%_ …, GTV_90%_ were constructed based on the CT images corresponding to the ten breathing phases obtained using the retrospective gating method and were merged into IGTV_retro_. Based on IGTV_pro_ and IGTV_retro_, the subclinical lesion area was expanded to construct internal target volume (ITV_pro_) and ITV_retro_. The systematic error and setup error was further increased by 3 mm to form PTV_pro_ and PTV_retro_. The displacement of the tumor in the respiratory cycle under prospective gating was defined by comparing the difference in the center positions of the GTV_in_ and GTV_ex_. Compared to GTV_0%_, GTV_10%_ …, GTV_90%_ in the different respiratory phases, the maximum difference in the center position of GTV_0%_, GTV_10%_ … GTV_90%_ is the tumor displacement under a retrospective gating simulation. GTV_static_ was constructed on breath‐holding scan images, which represent the relatively true volume of the tumor. Radiotherapy plan, Plan_con_, Plan_pro_, and Plan_retro_, were designed based on PTV_con_, PTV_pro_, and PTV_retro_, respectively. The prescription dose of the PTV was 6000 cGy in all three plans. V_5Gy_, V_10Gy_, V_20Gy_, and V_30Gy_, and mean lung doses for the affected lung and bilateral lung were calculated in the three plans.

### 2.4 Statistical analysis

Statistical analysis was performed using SPSS software (version 19.0; IBM SPSS Inc., Chicago, IL, USA). Data distribution was pretested for normality and homogeneity of variance. The comparison of different IGTVs, ITVs, PTVs, and tumor center displacements based on the two respiratory gating methods was performed using the Wilcoxon signed rank sum test, and the dosimetry parameters in the radiotherapy plan were compared. Differences were considered statistically significant at a *p‐*value <0.05.

## 3 RESULTS

### 3.1 Comparison of tumor center displacements

There was no significant difference in tumor center displacement measured using the prospective and retrospective respiratory gating methods (Table 1). Both methods showed that the displacement in the superior–inferior direction was greater than those in the left–right and anterior–posterior directions.

**TABLE 1:** Comparison of tumor center displacements based on two respiratory gating methods (unit: mm).

Gating method	Group A			Group B		
	X (Lateral)	Y (AP)	Z (CC)	X (Lateral)	Y (AP)	Z (CC)
Prospective	0.83 ± 0.63	0.80 ± 0.82	1.82 ± 2.06	0.99 ± 0.64	1.89 ± 2.63	6.69 ± 5.67
Retrospective	0.91 ± 0.65	1.22 ± 1.04	1.77 ± 1.88	1.08 ± 0.64	2.43 ± 2.76	7.92 ± 5.78
*Z*	0.134	1.482	0.060	0.098	1.432	1.299
*p*	0.893	0.138	0.952	0.922	0.155	0.194

### 3.2 Comparison of PTVs based on different simulation methods

**FIGURE 1:**
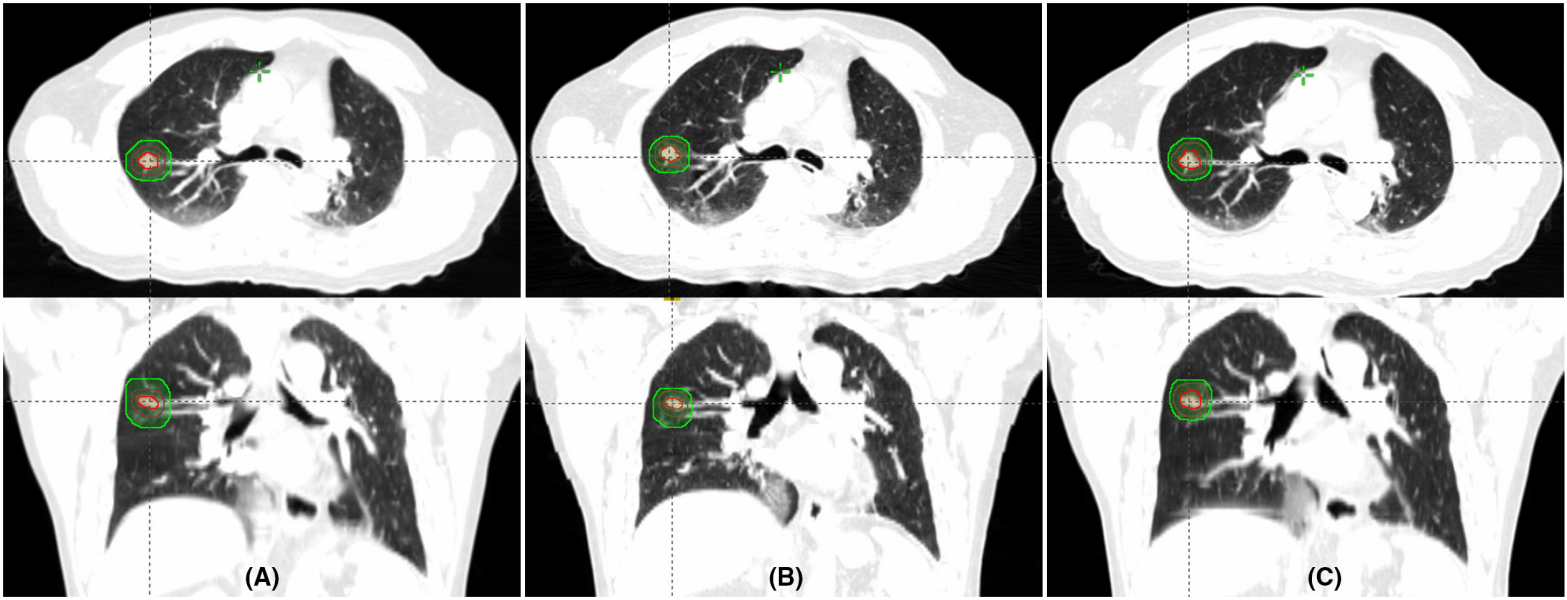
Planning target volumes based on different simulation methods. (A) 3D no gating simulation; (B) prospective gating simulation; (C) retrospective gating simulation.

For all patients (group A + B), PTV_con_ was 59.02 ± 4 5.62 cm^3^. PTV_pro_ was 35.01 ± 27.85 cm^3^. PTV_retro_ was 39.89 ± 30.77 cm^3^ (Figure 1). PTV_pro_ and PTV_retro_ were both lesser than PTV_con_ (*p* < 0.001). A statistical difference in the volume was also observed between PTV_pro_ and PTV_retro_ (*p* < 0.05) (Table 2).

**TABLE 2:** Comparison of tumor target volumes based on two respiratory gating methods (unit: cm^3^).

Gating method	Group A			Group B		
	IGTV	ITV	PTV	IGTV	ITV	PTV
Prospective	5.51 ± 4.55	18.74 ± 12.28	32.92 ± 18.52	6.83 ± 11.37	21.72 ± 24.25	36.78 ± 34.54
Retrospective	5.78 ± 4.65	20.47 ± 12.13	37.13 ± 18.75	8.13 ± 14.67	24.39 ± 27.88	42.22 ± 38.84
*Z*	2.045	2.134	2.667	2.237	2.062	2.904
*p*	0.041	0.033	0.008	0.025	0.039	0.004

Abbreviations: IGTV, internal gross tumor volumes; ITV, internal target volumes; PTV, planning target volumes.

### 3.3 Comparison of dosimetry parameters in the radiotherapy plans

**FIGURE 2:**
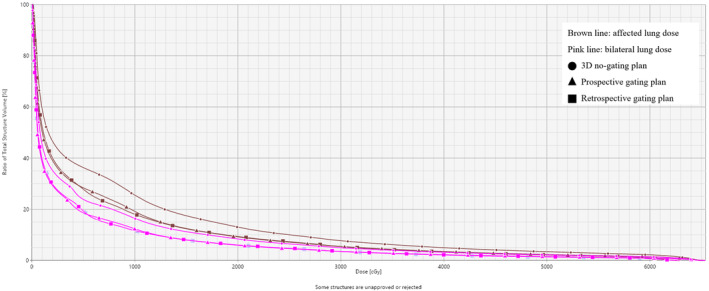
Radiation doses to normal lung tissues based on different simulation methods.

The dosimetry of V_5Gy_, V_10Gy_, V20_Gy_, V30_Gy_, and mean dose to the affected lung/bilateral lung in Plan_con_ were 24.35 ± 9.16/38.39 ± 9.50, 14.12 ± 5.27/27.96 ± 9.09, 8.02 ± 3.17/16.82 ± 7.66, 5.22 ± 2.99/11.00 ± 6.47, and 558.0 ± 208.94/994.02 ± 365.83 (cGy), respectively. The radiation doses were higher than those in Plan_pro_ and Plan_retro_ (both *p* < 0.001) (Figure 2). However, there was no significant difference in V_5Gy_, V_10Gy_, V20_Gy_, V30_Gy_ and mean dose to the affected lung/bilateral lung between Plan_pro_ and Plan_retro_ (*p* > 0.05) (Table 3).

**TABLE 3:** Comparison of dosimetry parameters using different gating methods.

Plan	Radiation dose to affected lung					Radiation dose to bilateral lung				
	V_5Gy_ (%)	V_10Gy_ (%)	V_20Gy_ (%)	V_30Gy_ (%)	Mean (cGy)	V_5Gy_ (%)	V_10Gy_ (%)	V_20Gy_ (%)	V_30Gy_ (%)	Mean (cGy)
Plan_pro_	31.13 ± 9.61	21.61 ± 8.41	12.57 ± 6.25	8.01 ± 4.54	773.13 ± 293.95	19.01 ± 7.36	10.71 ± 4.51	5.93 ± 3.00	3.61 ± 2.06	435.89 ± 168.47
Plan_retro_	31.33 ± 8.95	21.84 ± 7.74	12.73 ± 6.28	8.13 ± 4.75	770.35 ± 307.50	19.44 ± 6.98	10.94 ± 4.32	6.06 ± 3.03	3.75 ± 2.14	451.75 ± 234.51
*Z*	0.186	0.629	0.429	0.014	0.200	0.682	0.868	0.700	0.325	0.129
*p*	0.853	0.530	0.668	0.989	0.841	0.495	0.386	0.484	0.745	0.898

### 3.4 Comparison of movement information included in IGTV

The ratio of IGTV_pro_ to GTV_static_ constructed during breath‐holding was 1.41 ± 0.39. IGTV_retro_/GTV_static_ was 1.52 ± 0.52. Both IGTV_pro_ and IGTV_retro_ largely included tumor motion information. Furthermore, the ratio of IGTV to GTV_static_ was greater in group B than in group A (Table 4).

**TABLE 4:** Comparison of tumor motion information in IGTV_pro_ and IGTV_retro_ (namely the IGTV to GTV_static_ ratio).

Group	Prospective	Retrospective
Group A	1.28 ± 0.25	1.36 ± 0.22
Group B	1.65 ± 0.32	1.77 ± 0.51
*Z*	2.608	2.550
*p*	0.007	0.009

Abbreviations: GTV, gross tumor volume IGTV, internal gross tumor volumes.

## 4 DISCUSSION

The application of prospective and retrospective respiratory gating technology in radiotherapy simulation allows the measurement of the 3D displacement of thoracic and abdominal tumors affected by respiratory motion.^17^ In this study, the displacements of SPTs measured using prospective and retrospective gating methods were compared, and the results were consistent with those of previous studies.^8^, ^18^ Li et al.^9^ showed that the displacement amplitudes of lung tumors in the left–right, anterior–posterior, and superior–inferior directions were 1.6 mm, 2.2 mm, and 5.5 mm, respectively, which were within the range of displacement amplitudes observed in this study. Prospective gating involves the collection of position and shape information of the tumor at two specific respiratory states (end‐inspiration and end‐expiration) and measures the difference in tumor center position between these two states. Retrospective gating was used to determine the movement trajectory of the tumor throughout the entire respiratory cycle. Based on the patient's respiratory waveform, the entire breathing cycle was divided into ten respiratory phases. The maximum difference in tumor center displacement among the ten phases was then measured.^19^ Both gating methods involved simulation and image acquisition during calm breathing. While the two approaches differ with respect to the image acquisition methods (axial vs. helical), the respiratory phases used to acquire the tumor position and shape information were similar. Therefore, the tumor center displacement measured using the two gating methods had no significant difference.

Respiratory gating technology facilitates the measurement of SPT displacement individually, and there is no significant difference in the results obtained using the two gating methods. In this study, we delineated the IGTV and analyzed tumor motion information based on prospective gating and retrospective gating. The results showed that the volume of IGTV_pro_ delineated using prospective gating was lesser than IGTV_retro_ that delineated on the retrospective gating images. In patients with tumors located in the upper lobes in group A, IGTV_pro_ was lesser than IGTV_retro_ in nine cases and greater than IGTV_retro_ in two cases. In group B, which included patients with tumors located in the middle or lower lobes, IGTV_pro_ was lesser than IGTV_retro_ in ten cases and greater than IGTV_retro_ in three cases. In most tumors, IGTV_retro_ delineated on the retrospective gating images provided more tumor motion information than that obtained using the prospective gating method. In IGTV_pro_, based on prospective gating, only the position and shape information of the end‐inspiration and end‐expiration phases was combined, which was insufficient to capture all movement information throughout the respiratory cycle. Specifically, this information cannot fully account for the overall displacement information in the 3D space.^18^, ^20^ IGTV_retro_, based on retrospective gating, included not only the 3D displacement and deformation information in the two states mentioned but also the trajectory of tumor movement during the respiratory cycle.^16^ The retrospective gating method provided more tumor movement information, which led to IGTV_retro_ being greater than IGTV_pro_. However, there remained five cases in which IGTV_pro_ was marginally greater than IGTV_retro_. The volume differences ranged from 0.18 cm^3^ to 0.41 cm^3^. This could be attributed to an artifact of respiratory movement or an error in target delineation.

IGTV_pro_ and IGTV_retro_ both provided information about tumor movement during the respiratory cycle, especially the tumor located in lower lobe. However, IGTV_pro_ and IGTV_retro_ were different in volume. Since the PTVs were derived by expanding subclinical lesions and accounting for setup errors based on IGTV, the PTVs based on the two methods also differed. The absolute difference in volume between PTV_pro_ and PTV_retro_ was more pronounced than the difference between IGTV_pro_ and IGTV_retro_. Nevertheless, the volumes of PTV_pro_ and PTV_retro_ were both significantly lesser than that of PTV_con_, which was constructed based on non gating methods. Individualized tumor target volumes constructed using gating techniques could significantly reduce the irradiation volume of normal lung tissue compared with that obtained using non gating approaches.^21^

Tumor displacements caused by respiratory motion have a significant influence on the target dose and dose distribution to OAR.^22^, ^23^ Radiotherapy plans, Plan_con_, Plan_pro_, and Plan_retro_ were designed based on PTV_con_, PTV_pro_, and PTV_retro_, respectively. PTV_con_, constructed using traditional non gating simulation, had a considerably larger volume than PTV_pro_ and PTV_retro_ based on the gating methods. An increase in PTV inevitably prolongs radiotherapy duration and increases the irradiation dose in normal lung tissues. In non gating radiotherapy plans, the V_5Gy_, V_10Gy_, V20_Gy_, V30_Gy_, and mean dose for normal lung tissue in Plan_con_ were significantly higher than those in Plan_pro_ and Plan_retro_. Although IGTV_retro_ and PTV_retro_, constructed based on the retrospective respiratory gating method, were larger than IGTV_pro_ and PTV_pro_, dosimetry comparison revealed that the doses for PTV_pro_ and PTV_retro_ were 6315.6 ± 21.12 cGy and 6310.54 ± 26.53 cGy, respectively. There was no significant difference between the two plans with respect to the radiation dose for the PTV (*p* = 0.599). Moreover, the difference in V_5Gy_, V_10Gy_, V20_Gy_, V30_Gy_ and mean dose between plan_pro_ and plan_retro_ was not significant. This may be attributed to the volume percentage of the PTV in the affected and bilateral lungs. The volume percentages of PTV_pro_ and PTV_retro_ in the affected lung were 2.48% and 2.75% and in the bilateral lung were 1.15% and 1.28%, respectively. Given that the PTV occupies a small percentage of the affected lung and the bilateral lung volume, the two volume percentages were considerably close, with only a marginal difference of less than 0.5%. Therefore, there was no statistical difference in the V_5Gy_, V_10Gy_, V20_Gy_, V30_Gy_, and mean dose of the affected lung/bilateral lung in the radiotherapy plans based on the two gating methods. In comparison with the traditional non gating 3DCT simulation, the gating method could significantly reduce the irradiation dose to normal lung tissue. IGTV_retro_ and PTV_retro_, based on retrospective respiratory gating technology, provided more tumor movement information than IGTV_pro_ and PTV_pro_, whereas Plan_retro_ did not increase the irradiation dose on normal lung tissue significantly.^24^

Respiratory gating technology enables the accurate assessment of SPT displacement in the respiratory cycle, facilitating individualized target volume delineation and radiotherapy plan design. Gating radiotherapy plans can reduce the irradiation dose to normal lung tissues and minimize radiation‐induced lung injury, while ensuring target coverage and dose delivery. Compared to prospective respiratory gating technology, PTVs based on retrospective gating technology provide additional tumor movement information without significantly increasing the irradiation dose in normal lung tissues.

## AUTHOR CONTRIBUTIONS

**Dongping Shang:** Conceptualization (equal); data curation (equal); formal analysis (equal); resources (equal); writing – original draft (equal); writing – review and editing (equal). **Jinghao Duan:** Data curation (equal); formal analysis (equal); resources (equal); validation (equal). **Yong Yin:** Project administration (lead); supervision (lead); writing – review and editing (equal). **Ruozheng Wang:** Data curation (equal); validation (equal); visualization (equal).

## FUNDING INFORMATION

This research was supported by Shandong medical science and technology development project (Grant No. 202109030501) and National Nature Science Foundation of China (Grant Nos. 82,072,094, 12,275,162).

## CONFLICT OF INTEREST STATEMENT

The authors have no conflicts of interest.

## Data Availability

Raw data were generated at Shandong Cancer Hospital and Institute. Derived data supporting the findings of this study are available from the corresponding author Shandong Cancer Hospital and Institute on request.
